# Latent Class Analysis to Explore Subtypes of EGPA: Focusing on Respiratory Involvement and Inflammation Markers

**DOI:** 10.5152/ArchRheumatol.2025.25095

**Published:** 2026-04-03

**Authors:** Yu Gu, Yitong Liu, Ting Zhang, Yang Han, Min Peng, Juhong Shi

**Affiliations:** 1 Department of Pulmonary and Critical Care Medicine, Capital Medical University, Beijing Anzhen Hospital, Beijing, China; 2 Department of Pulmonary and Critical Care Medicine, Chinese Academy of Medical Science & Peking Union Medical College, Peking Union Medical College Hospital, Beijing, China; 3 Department of Infectious Diseases, Chinese Academy of Medical Science & Peking Union Medical College, Peking Union Medical College Hospital, Beijing, China

**Keywords:** Anti-neutrophil cytoplasmic antibody, eosinophilic granulomatosis with polyangiitis, latent class analysis

## Abstract

**Background/Aims:**

Eosinophilic granulomatosis with polyangiitis (EGPA) was regarded as a heterogeneous disease with respiratory involvement manifested in various patterns. This study aimed to explore whether subtypes with different clinical features and outcomes could be identified by latent class analysis (LCA) from the perspective of the respiratory system in patients with EGPA.

**Materials and Methods:**

Patients diagnosed with EGPA between January 2000 and December 2022 were included. The clinical data and survival of the individuals were collected. Subtypes were identified using LCA according to organ involvement and anti-neutrophil cytoplasmic antibody (ANCA) status in model 1, according to patterns of respiratory involvement, other organ involvement, ANCA status, and inflammatory markers in model 2. The characteristics and prognosis of the classes were compared.

**Results:**

Out of the 330 patients initially diagnosed with EGPA, 245 patients were included eventually. In model 1, 138 (56.3%) and 107 (43.7%) patients were identified in classes 1 and 2, respectively. Class 2 was older (*P* = .017), had less musculoskeletal, mucocutaneous, cardiovascular, gastrointestinal, and peripheral nervous involvement (all *P* < .001) compared to class 1. No significant difference in overall survival was found between the classes. In model 2, LCA assigned 165 (67.3%) participants to class 1 (systemic EGPA) and 80 (32.7%) to class 2 (respiratory-limited EGPA). Compared with class 1, class 2 was younger (*P* < .001) and had lower levels of inflammation markers (white blood cell, eosinophil, erythrocyte sedimentation rate, and C-reactive protein: *P* < .001). Besides, patients in class 2 were more likely to have airway involvement, including the onset of asthma (*P* = .041), FEV1/FVC < 70% (*P* < .001), and bronchiectasis (*P* < .001). Moreover, class 2 exhibited better survival compared to class 1 (*P* = .001).

**Conclusion:**

The respiratory-limited EGPA may be the relatively milder type of disease. Other organ functions, serum inflammatory markers, and ANCA should be monitored during follow-up in these patients.

**Main Points**

- The patients with eosinophilic granulomatosis with polyangiitis (EGPA) could be grouped into the systemic and respiratory-limited ones by organ involvement, patterns of respiratory involvement, anti-neutrophil cytoplasmic antibody (ANCA) status, and inflammation markers using latent class analysis.
- The patients with respiratory-limited EGPA, which was probably the relatively milder subtype of EGPA, had a lower level of inflammation and better prognosis.
- It was necessary to monitor the other organ functions, serum inflammatory markers, and ANCA during follow-up of EGPA.

## Introduction

Eosinophilic granulomatosis with polyangiitis (EGPA) is one of the anti-neutrophil cytoplasmic antibody (ANCA)-associated vasculitis (AAV), characterized by refractory asthma, eosinophil infiltration, and granuloma formation.^1^ Eosinophilic granulomatosis with polyangiitis has heterogeneity and variability in clinical manifestations. A previous study found that EGPA patients with positive ANCA had higher risks of peripheral nervous, cutaneous, and renal involvement, but lower risks of pulmonary infiltrates and cardiac involvement.^2^ On pathology, 2 distinct subtypes were classically described based on ANCA status: the ANCA-negative subset with eosinophil infiltration and the ANCA-positive one with small-to-medium vasculitis.^3^ However, in a recent study, Rubenstein et al^4^ performed a cluster analysis of 489 patients with EGPA according to organ involvement and ANCA status but did not reveal clinically meaningful subgroups. There was a lack of other analyses on classification due to the rarity of EGPA.

Eosinophilic granulomatosis with polyangiitis involving the respiratory system could present with a variety of manifestations, such as asthma, bronchiectasis, pulmonary infiltrates, or hemorrhage.^5^ Eosinophilic granulomatosis with polyangiitis classically develops into 3 consecutive phases.^6^ Patients usually initiate with asthma and allergic rhinosinusitis (prodromal phase). After 9.3 ± 10.8 years, they may gradually develop pulmonary infiltrates, eosinophilic cardiomyopathy, and/or gastroenteritis (eosinophilic phase).^7^ Subsequently, some patients present with necrotizing vasculitis, such as glomerulonephritis, mononeuropathy, and pulmonary hemorrhage (vasculitic phase). This evolution suggests that the respiratory system is involved throughout the course of the disease. Therefore, it is of great importance to consider the diversity of respiratory involvement when classifying EGPA patients.

Erythrocyte sedimentation rate (ESR) and C-reactive protein (CRP) played an important role in evaluating the prognosis of AAV patients. The cluster analysis of the Japanese AAV population found that CRP was associated with important organ involvement, except for renal.^8^ Moreover, clusters separated by CRP and creatinine levels predicted the prognosis of overall, ESRD end-stage renal disease-free, and relapse-free survival rates.^8^ A previous study by the authors also found that ANCA-positive idiopathic interstitial pneumonia patients with increased ESR or CRP had a worse prognosis than those with normal inflammatory markers.^9^

A previous study focused on the impact of organ involvement and ANCA status on the clinical subtypes of EGPA.^4^ While another study also analyzed the various patterns of respiratory involvement in EGPA specifically.^5^ However, there was no study that combined the specific manifestations of respiratory involvement with the overall manifestations for analysis. The patients with EGPA usually visit the respiratory department first, as respiratory symptoms are the dominant manifestations during the early phase. Thus, pulmonologists should identify those with a high risk of poor prognosis among the EGPA patients with respiratory involvement. The study aims to identify subtypes of EGPA according to common organ involvement, patterns of respiratory involvement, ANCA status, and inflammatory markers using latent class analysis (LCA) to explore whether the clinical characteristics and outcomes of the subtypes are different.

## Materials and Methods

### Selection of Patients

Eligible patients had a physician-confirmed diagnosis of EGPA between January 2000 and December 2022. All consecutive cases diagnosed as EGPA were reassessed by the investigators. Patients who were diagnosed with vasculitis and fulfilled the American College of Rheumatology (ACR) classification criteria for EGPA^10^ and/or the criteria used by Cottin et al^11^ were included. The following patients were excluded: 1) under 18 years old; 2) complicated with malignancies, infectious diseases, or other autoimmune diseases; and 3) complicated with other hypereosinophilic syndromes. The diagnosis of patients was independently assessed by 2 physicians (Y.G. and Y.L.), and checked by an experienced pulmonologist (J.H.) to resolve any disagreements. The baseline data of the patients before receiving treatment were collected. Treatment was left to the discretion of the physician and followed the standard of care guidelines. All patients were followed until February 2024 or death. The study complied with the Declaration of Helsinki and was approved by Peking Union Medical College Hospital (Approval No. I-22PJ1119, Approval Date: December 29, 2022). Written informed consent was obtained from the patients who agreed to take part in the study.

### Data Collection and Definitions

Demographic data, organ involvement, laboratory findings, pathological findings, treatments, and outcomes were collected. The five-factor score (FFS) was calculated to assess the risk of prognosis.^12^ Ten domains of organ involvement, including musculoskeletal, mucocutaneous, ophthalmologic, ear-nose-throat (ENT), respiratory, cardiovascular, gastrointestinal, renal, peripheral, and central nervous system involvement, were defined according to the study of Rubenstein et al.^4^ The sequences of organ involvement in the patients and the initial diagnosis of the first visit were recorded. Asthma was diagnosed according to the 2022 Global Strategy for Asthma Management and Prevention (GINA). The thresholds used to define elevated ESR were 15 mm/h for males and 20 mm/h for females, while for CRP, it was 10 mg/L. The ratio of forced expiratory volume in 1 second to forced vital capacity (FEV1/FVC) < 70% was considered to have obstructive ventilatory dysfunction. Bronchiectasis, ground glass opacity, bronchovascular bundle thickening, and consolidation were evaluated following the Fleischner Society criteria.^13^ The ground glass opacity, bronchovascular bundle thickening, and consolidation were regarded as pulmonary infiltration.^14^ Chest computed tomography images of the included patients were independently reviewed by 2 experienced pulmonologists (T.Z. and M.P.), who were blinded to the clinical data. Any discrepancies were resolved through discussion with a more experienced pulmonologist (J.S.). Pulmonary hemorrhage was defined by the clinical trial of the PEXIVAS study.^15^ The pathology was evaluated by 2 pathologists separately. Any disagreements were resolved through discussion within the department.

### Latent Class Analysis

**Figure 1.**
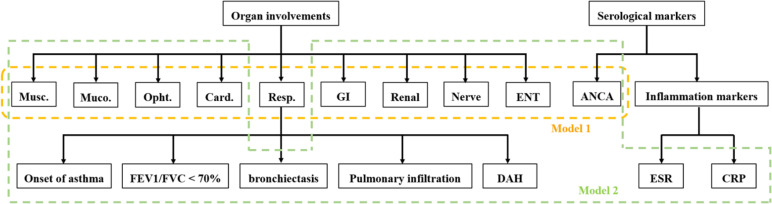
The variables included in the latent class analysis of model 1 and model 2.

Latent class analysis is an algorithm used to split individuals into smaller subgroups with shared characteristics.^16^ Supplementary Table 1 provided prevalence distributions for all variables. The variables with a prevalence of less than 5% were excluded, such as central nervous system involvement (n = 2, 0.65%). According to the cluster analysis model of Rubenstein et al,^4^ model 1 considered 10 categorical input variables, including musculoskeletal, mucocutaneous, ophthalmologic, ENT, respiratory, cardiovascular, gastrointestinal, renal, peripheral nervous system involvement, and ANCA status. Model 2 included inflammation markers (increased ESR or CRP) as additional input variables and substituted specific manifestations (onset of asthma, FEV1/FVC < 70%, bronchiectasis, pulmonary infiltration, and pulmonary hemorrhage) for respiratory involvement, to explore whether the inflammation status and patterns of respiratory involvement could affect the formation of subgroups (Figure 1).

In LCA, a series of models was estimated, starting with a 1-class model and incrementally increasing the number of classes until a model with 4 classes was reached. Due to the small size, models consisting of 5 or more classes were not considered. Specifically, 100 random sets of starting values were used for the initial stage of estimation and 20 optimizations for the final stage to verify that the log-likelihood was replicated and that the global maximum was found, rather than a local solution. The best-fitting model using entropy determined the Bayesian information criteria, Vuong–Lo–Mendell–Rubin likelihood-ratio test, and proportions of each class. While statistical indices guided the selection, the final choice of the optimal class solution was primarily based on interpretability and clinical relevance, ensuring that the derived classes were meaningful and distinct within the study context. Finally, each patient was grouped into a class according to the model-generated probabilities. The item-response probabilities profile was examined for each latent class to ensure that each class represented a distinct and clinically coherent pattern. The accuracy of the class assignment was assessed by reviewing the average posterior class assignment probabilities. High diagonal values and low off-diagonal values in the matrix of average posterior probabilities indicate that individuals were classified with a high degree of certainty into their most likely class. Mplus version 8.3 (Muthén & Muthén) was used to perform LCA.

### Other Statistical Analysis

SPSS version 26.0 (IBM SPSS Corp.; Armonk, NY, USA) was used to perform analyses. Continuous data were exhibited as mean with SD or median with interquartile range. Categorical data were presented as numbers with percentages. Tests such as *t*-tests, Mann–Whitney *U*-tests, or chi-square tests were used as appropriate to assess the differences in mean, median, and proportion between classes. Inter-rater reliability was calculated using the kappa statistic. The Kaplan–Meier method, Cox regression, and log-rank tests were used to analyze survival data. Two-tailed *P* < .05 was considered significant.

## Results

### Patients’ Characteristics

**Figure 2.**
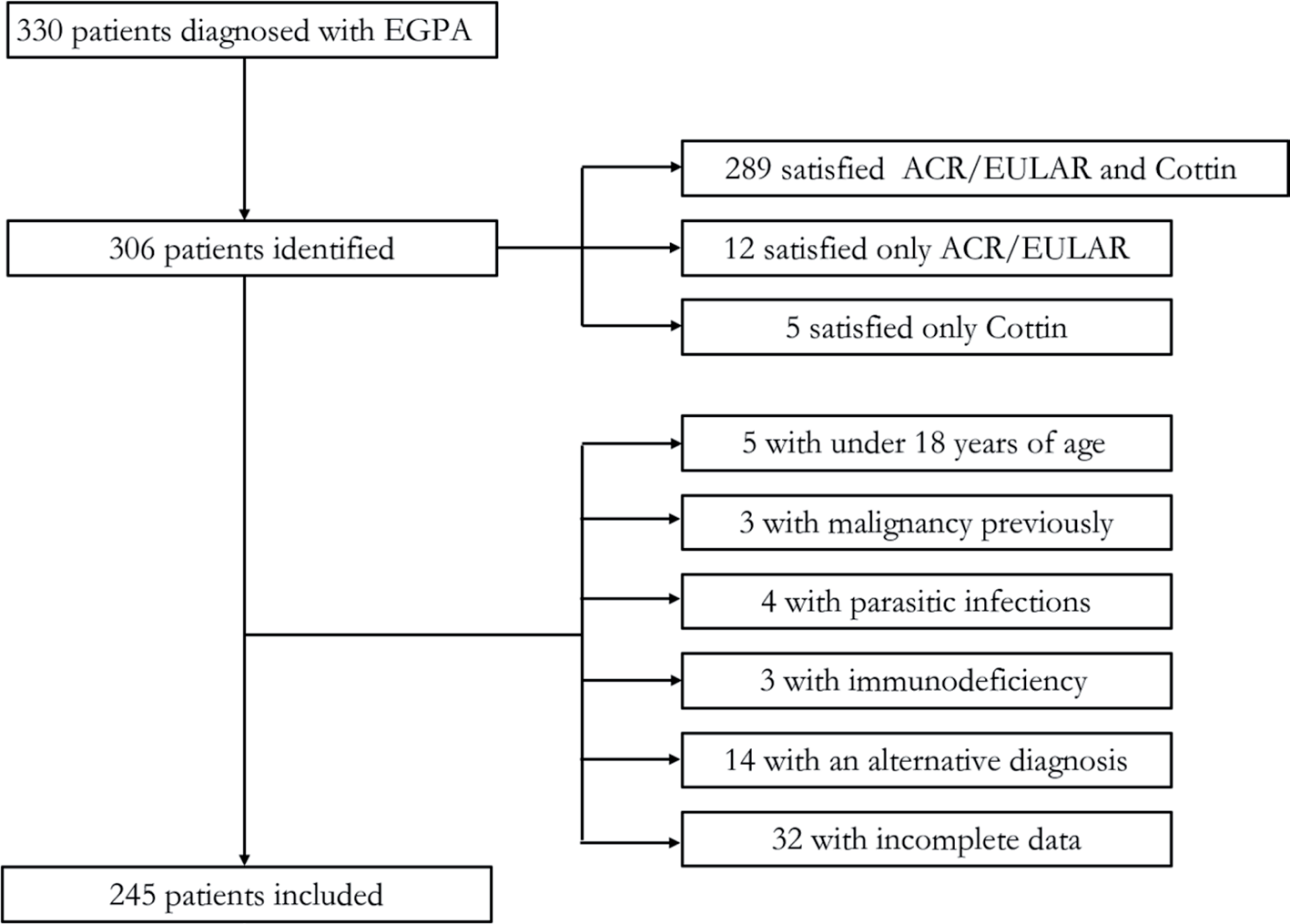
Flowchart of the included eosinophilic granulomatosis with polyangiitis and ACR/EULAR, American College of Rheumatology/European League Against Rheumatism (EGPA) patients.

A total of 330 patients were diagnosed with EGPA upon discharge. According to their clinical data, 289 patients satisfied both the ACR/EULAR 2022 criteria and Cottin criteria simultaneously, while 12 patients met the ACR/EULAR 2022 criteria and 5 patients met the Cottin criteria. Of the 306 consecutive patients, 61 were excluded for the following reasons: 5 were under 18 years of age; 3 had malignancies, 4 had parasitic infections, 3 had immunodeficiency; 14 were ultimately diagnosed with other diseases; and 32 had incomplete data. The patient selection process is outlined in Figure 2. The mean age of the patients was 58 years, and 59.2% of them were male. The most frequently observed organ involvements were respiratory (95.1%), ENT (73.9%), peripheral nervous system (61.2%), cardiovascular (60.8%), and gastrointestinal (53.1%). Among the 51 ANCA‑positive patients, 2 were positive for PR3‑ANCA, 42 were positive for MPO‑ANCA, and 7 were concurrently positive for both PR3‑ANCA and MPO‑ANCA. The mean titer of PR3‑ANCA was 108 ± 52 IU/L, while that of MPO‑ANCA was 115 ± 38 IU/L. The Supplementary Table 2 showed that the agreement between independent radiology/pathology findings by 2 readers was greater for all items (κ > 0.75). The follow-up time was 4723 (3292-6375) days, and 13.1% of patients died during this period.

### Latent Class Analysis in Model 1

Latent class analysis was performed based on the 10 variables in 245 patients with EGPA. According to the Supplementary Table 3, the best-fit model was the 2-class Model 1. The patients in class 1 had high probabilities of organ involvement, including respiratory, peripheral nervous, cardiovascular, ENT, mucocutaneous, and musculoskeletal systems. However, the patients in class 2 were only likely to have respiratory and ENT involvement (Table 1).

**Figure 3.**
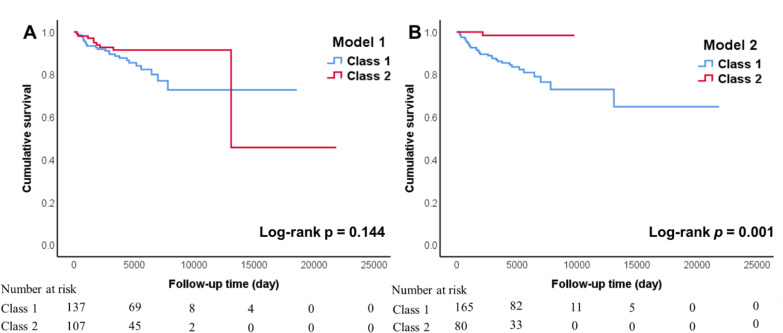
Kaplan–Meier survival curves comparing overall survival between class 1 and class 2 in model 1 (A) and model 2 (B).

The characteristics of 138 (class 1, 56.3%) and 107 (class 2, 43.7%) patients from the 2 classes, respectively, are displayed in Supplementary Table 4. Compared with class 1, patients in class 2 were older (*P* = .017), had a lower FFS (*P* = .007), less musculoskeletal (*P* < .001), mucocutaneous (*P* < .001), cardiovascular (*P* < .001), gastrointestinal (*P* < .001), and peripheral nervous (*P* < .001) involvement. In laboratory findings, class 1 had significantly increased inflammation markers, such as white blood cell (WBC) counts (*P* < .001), ESR (*P* = .026), CRP (*P* < .001), and eosinophil counts (*P* < .001). Moreover, class 1 was more likely to find vasculitis in pathology (*P* = .015), while class 2 was characterized by eosinophilic infiltration (*P* = .002). Comparisons of the treatments showed statistically significant differences in the frequency of using cyclophosphamide (*P* = .010) and azathioprine (*P* = .003) as well. During the follow-up time of 4723 (interquartile range [IQR]: 3292-6375) days, the significant difference in overall survival was not found between class 1 (83.3%) and class 2 (91.6%, Figure 3A).

### Latent Class Analysis in Model 2

Next, to elucidate the common patterns of respiratory involvement and the inflammatory markers that could affect the classification of EGPA, respiratory involvement was replaced in Model 2 of the LCA by the onset of asthma, FEV1/FVC < 70%, bronchiectasis, pulmonary infiltration, and pulmonary hemorrhage. Additionally, increased ESR and CRP were added to model 2 of LCA. Similar to model 1, the 2-class model was considered the best fit for model 2 (Supplementary Table 5). In class 1, the probabilities of cardiovascular, peripheral nervous, ENT, gastrointestinal, mucocutaneous, and musculoskeletal involvements were more than 50%. While class 2 only had a high probability of ENT involvement. In the respiratory system, both class 1 and class 2 had a higher probability of pulmonary infiltration. However, the patients in class 2 were prone to present with asthma initially, had bronchiectasis, and obstructive ventilation dysfunction. In addition, the patients in class 1 had more than 70% probabilities of having elevated ESR and CRP (Table 1).

**Figure 4.**
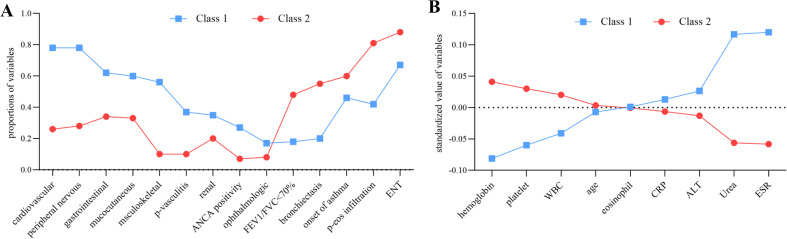
The comparisons of clinical parameters between class 1 and class 2 in model 2.

The model 2 assigned 165 (67.3%) patients to class 1 and 80 (32.7%) patients to class 2. The comparisons of the 2 classes are shown in Table 2. The patients in class 1 were older (*P* < .001) with a higher FFS (*P* < .001) compared with class 2. While class 2 was more likely to have airway involvement, including the onset of asthma (45.5% vs. 60.0%, *P* = .041), FEV1/FVC < 70% (18.2% vs. 47.5%, *P* < .001), and bronchiectasis (20.0% vs. 55.0%, *P* < .001). However, except for ENT and respiratory involvement, other organ involvement in class 1 was significantly higher than that in class 2 (*P* < .05). Like model 1, class 1 had higher levels of inflammation markers (WBC, eosinophil, ESR, and CRP: *P* < .001), more vasculitis, but less eosinophil infiltration in pathology. More importantly, more than one-fourth of patients in class 1 had ANCA positivity, which was significantly higher than that in class 2 (27.3% vs. 7.5%, *P* < .001). The differences in organ involvement, patterns of respiratory involvement, pathological findings, and ANCA positivity between class 1 and class 2 are illustrated in Figure 4A. The comparisons of laboratory findings are shown in Figure 4B. Besides, the differences in the frequency of treatment with cyclophosphamide (*P* < .001) and azathioprine (*P* < .001) could also be seen in model 2. In the survival analysis, class 2 showed a better survival rate compared to class 1 (*P* = .001, Figure 3B). In Cox regression, the patients of class 2 also had a decreased risk of death (*P* = .02, hazard ratio [HR] = 0.094 with C.I. of 0.013-0.693 adjusted for gender and age).

## Discussion

Latent class analysis was conducted using common organ involvements, patterns of respiratory involvements, ANCA status, and inflammation markers in patients with EGPA. In model 2, 2 distinct subtypes with different clinical features and survivals were identified. The class 1, referred to as “EGPA with systemic involvements,” was characterized by high frequencies of organ involvement, increased inflammation markers, and ANCA positivity. The class 2, named “respiratory-limited EGPA,” was likely to have ENT and airway involvement with negative ANCA and normal-range inflammation markers.

Previous studies have proposed various classification methods for EGPA patients by different scholars. Given the strong association between ANCA positivity and vasculitis, ANCA status was usually used to classify EGPA.^2^^,^^11^ However, Rubenstein et al^4^ recently obtained 5 subtypes with overlapping clinical features using cluster analysis according to organ involvement and ANCA status. Thus, they suggested that EGPA should be regarded as a phenotypic spectrum rather than a dichotomous disease.^4^ Eosinophil infiltration is also a dominating feature of EGPA, except for vasculitis.^6^^,^^7^^,^^17^ Cottin et al^11^ demonstrated that not all the patients with a clinical diagnosis of EGPA had vasculitis and proposed a new entity referred to as hypereosinophilic asthma with systemic to define the population only with eosinophilic proliferation Recently, another study illustrated that blood eosinophil ≤ 1500 cells/mL was predictive of asthma, ENT involvement, and pulmonary infiltration, while blood eosinophil > 3500/mL was predictive of extrapulmonary organ involvement.^18^ Subsequently, they proposed 2 EGPA phenotypes, named systemic and respiratory-limited EGPA, which were consistent with the present results.^18^ In model 2, the 3-class solution split patients with systemic disease into 2 types. However, the average posterior probabilities decreased to 0.65, 0.61, and 0.87, illustrating that the characteristics of patients were blurred.

This study showed that the prognosis of EGPA patients should be evaluated by combining the involved organs, ANCA status, and inflammatory markers simultaneously. While extrapulmonary involvement alone was insufficient to predict the outcome. In this study, class 2 with prominent airway involvement probably represented a milder type of EGPA. While the patients in class 1 with extra-airway involvement, ANCA positivity, and increased inflammatory markers concurrently were at high risk of poor prognosis. Adult-onset refractory asthma was one of the typical manifestations of EGPA.^19^ Early identification of high-risk EGPA from severe asthma contributes to improving the prognosis. Although vasculitis is commonly recognized as a systemic disease, single-organ vasculitis should not be overlooked.^1^ According to the progression of “3 consecutive phases” of EGPA, severe eosinophilic asthma can progress to eosinophilic pulmonary disease or even to EGPA with vasculitis manifestations.^6^ In the study of Cottin et al,^11^ EGPA patients with a single systemic manifestation attributable to the disease, regarded as “formes frustes,” were included to account for early diagnosis. To date, there are no universally accepted criteria for EGPA.^6^ It is still difficult to identify patients at high risk of EGPA from severe eosinophilic asthma.^20^^,^^21^ In addition to pulmonary function tests and imaging, studies on samples from the respiratory tract should be given more importance. Pagnoux et al^22^ did not find a significant difference in over 50 serum cytokines and chemokines between patients with EGPA and asthma. However, a recent study found that sputum granulocyte-macrophage colony-stimulating factor and eosinophils might be useful biomarkers to support early diagnosis and treatment choices in severe eosinophilic asthmatics suspected of having EGPA.^23^

The levels of ESR and CRP were of great importance to assess the prognosis of EGPA patients. Previous studies showed that an increase in CRP levels before treatment of AAV was significantly associated with relapse and mortality.^24^^,^^25^ A cluster analysis of Japanese AAV patients suggested that the CRP level represented general, pulmonary, and neural symptoms but not renal symptoms.^8^ Another Asian study found that the ratio of ESR to CRP could not only estimate the disease activity but also predict all-cause mortality in AAV patients.^26^ Besides, this study also found that increased ESR and CRP were more common in class 2 of model 2 with systemic disease. Thus, it was reasonable to speculate that an increase in inflammatory markers during follow-up possibly indicated the evolution of the disease from the eosinophilic phase to the vasculitic phase. The ESR and CRP levels of patients with severe eosinophilic asthma should be closely monitored during follow-up to evaluate the disease progression early.^27^

The study has several limitations. Firstly, given the rarity of EGPA, the sample size of more than 20 years was still insufficient for a cluster analysis. It is more suitable for the rare disease to carry out cluster analysis in a multicenter study with a large sample size. Secondly, the patterns of pulmonary involvement of EGPA were not limited to the ones listed in this study. The other manifestations, such as small airway lesions (e.g., bronchiolitis or tree-in-bud sign), could also be seen in EGPA patients.^5^^,^^28^ In addition, the majority of patients initially presented with ENT symptoms, which may account for the small proportion of individuals who present with asthma. Thirdly, in this retrospective study, the majority of patients could only be followed up regarding survival status. However, the specific causes of death could not be traced, making it impossible to conduct cause-specific analysis. Fourthly, this study defined airflow obstruction using the fixed FEV1/FVC ratio of < 0.70, which is known to potentially overdiagnose obstruction in older adults and underdiagnose it in younger individuals. Besides, the exclusion of 32 patients due to incomplete data introduces the possibility of selection bias. To assess this potential bias, the available baseline characteristics (age and gender) between the included (n = 245) and excluded (n = 32) patients were compared. As presented in Supplementary Table 6, no significant differences were observed in these parameters, which somewhat reassures that the 2 groups were comparable. Lastly, dichotomizing continuous variables (ESR, CRP) could lead to a loss of statistical power and prognostic information. Latent class analysis model, including CRP, ESR, and eosinophil count as continuous variables, was analyzed. The results revealed that the EGPA patients could not be classified into latent classes (Supplementary Table 7). This finding suggested LCA models with continuous variables probably required larger sample sizes to achieve meaningful categories.

This is the first study that classified EGPA patients by LCA and evaluated the overall survival between the determined classes. After being clustered by organ involvement, patterns of respiratory involvement, ANCA status, and inflammation markers, 2 distinct subtypes of EGPA patients were obtained, termed systemic and respiratory-limited EGPA. The latter may be the relatively milder subtype of EGPA. It is necessary to closely monitor the other organ functions, serum inflammatory markers, and ANCA during follow-up. More intensive treatment strategies may delay the progression of the disease.

## Figures and Tables

**Table 1.** Probabilities of Variables in 2 Classes of Model 1 and Model 2

	**Model 1**		**Model 2**	
	**Class 1**	**Class 2**	**Class 1**	**Class 2**
Number	138	107	165	80
ANCA positivity	0.194	0.226	0.271	0.085
**System involvement**				
Musculoskeletal	0.622	0.127	0.55	0.13
Mucocutaneous	0.715	0.241	0.6	0.335
Ophthalmologic	0.138	0.139	0.165	0.087
ENT	0.722	0.761	0.684	0.845
Cardiovascular	0.808	0.346	0.765	0.302
Gastrointestinal	0.659	0.362	0.621	0.354
Renal	0.346	0.244	0.355	0.198
Peripheral nervous	0.83	0.326	0.765	0.315
Respiratory	0.934	0.973	–	–
**Patterns of respiratory involvement**				
FEV1/FVC < 70%	–	–	0.185	0.458
Pulmonary infiltration	–	–	0.748	0.686
Bronchiectasis	–	–	0.201	0.536
Pulmonary hemorrhage	–	–	0.103	0.052
Onset of asthma	–	–	0.454	0.596
**Inflammation markers**				
Elevated ESR	–	–	0.702	0.22
Elevated CRP	–	–	0.785	0.287

ANCA, anti-neutrophil cytoplasmic antibody; CRP, C-reactive protein; ENT, ear-nose-throat; ESR, erythrocyte sedimentation rate; FEV1/FVC, the ratio of forced expiratory volume in 1 second and forced vital capacity.

**Table 2.** The Characteristics of Patients in Class 1 and Class of Model 2

	**All**	**Class 1**	**Class 2**	***P***
Number	245	165 (67.3%)	80 (32.7%)	
Male, n (%)	145 (59.2)	101 (61.2)	44 (55.0)	.406
Age (years)	58 (43-70)	60 (45-72)	53 (39-63)	**<.001**
FFS	0.77 ± 0.82	0.94 ± 0.87	0.41 ± 0.59	**<.001**
**Respiratory involvements, n (%)**				
Onset of asthma	123 (50.2)	75 (45.5)	48 (60.0)	**.041**
FEV1/FVC < 70%	68 (27.8)	30 (18.2)	38 (47.5)	**<.001**
Bronchiectasis	77 (31.4)	33 (20.0)	44 (55.0)	**<.001**
Pulmonary infiltration	178 (72.7)	124 (75.2)	54 (67.5)	.224
Pulmonary hemorrhage	21 (8.5)	17 (10.3)	4 (5.0)	.225
**Other organ involvements, n (%)**				
Musculoskeletal	100 (40.8)	92 (55.8)	8 (10.0)	**<.001**
Mucocutaneous	125 (51.0)	99 (60.0)	26 (32.5)	**<.001**
Ophthalmologic	34 (13.9)	28 (17.0)	6 (7.5)	**.049**
ENT	181 (73.9)	111 (67.3)	70 (87.5)	**.001**
Cardiovascular	149 (60.8)	128 (77.6)	21 (26.3)	**<.001**
Gastrointestinal	130 (53.1)	103 (62.4)	27 (33.8)	**<.001**
Renal	74 (30.2)	58 (35.2)	16 (20.0)	**.018**
Peripheral nervous	150 (61.2)	128 (77.6)	22 (27.5)	**<.001**
**Laboratory findings**				
WBC (×10^9^/L)	10.8 (8.2-15.3)	12.6 (8.9-19.0)	9.1 (7.4-10.9)	**<.001**
Eosinophil (×10^9^/L)	3.0 (1.3-6.4)	3.9 (1.7-7.9)	1.8 (0.9-2.9)	**<.001**
Lymphocyte (×10^9^/L)	1.8 (1.4-2.5)	1.8 (1.3-2.6)	2.0 (1.6-2.5)	.263
Hemoglobin (g/L)	131 ± 23	124 ± 22	147 ± 14	**<.001**
Platelet (×10^9^/L)	256 (199-333)	257 (182-353)	254 (224-321)	.600
ALT (U/L)	21 (13-38)	23 (15-45)	17 (13-27)	**.004**
Urea (mmol/L)	5.0 (3.9-6.9)	5.3 (3.9-7.6)	4.6 (3.7-5.6)	**.007**
Creatinine (μmol/L)	73 (62-86)	73 (61-89)	72 (63-83)	.482
ESR (mm/h)	20 (7-51)	35 (12-67)	8 (4-14)	**<.001**
CRP (mg/L)	7.1 (1.8-37.3)	16.7 (4.1-59.8)	1.7 (0.5-4.4)	**<.001**
ANCA positivity, n (%)	51 (20.8)	45 (27.3)	6 (7.5)	**<.001**
**Pathological findings, n(%)**				
Vasculitis	44 (29.5)	40 (37.0)	4 (9.8)	**.001**
Granuloma	10 (6.7)	7 (6.5)	3 (7.3)	1.000
Eosinophil infiltration	78 (52.3)	45 (41.7)	33 (80.5)	**<.001**
**Treatments and outcomes**				
Cyclophosphamide, n (%)	162 (66.1)	124 (75.2)	38 (47.5)	**<.001**
Azathioprine, n (%)	25 (10.2)	7 (4.2)	18 (22.5)	**<.001**
Methotrexate, n (%)	19 (7.8)	14 (8.5)	5 (6.3)	.619
Mycophenolate mofetil, n (%)	12 (4.9)	6 (3.6)	6 (7.5)	.214
Follow-up time (d)	4723 (3292-6375)	5010 (3441-6505)	4495 (2546-6153)	.265
Death, n (%)	32 (13.1)	31 (18.8)	1 (1.3)	**<.001**

ALT, alanine transaminase; ANCA, anti-neutrophil cytoplasmic antibody; CRP, C-reactive protein; ENT, ear-nose-throat; ESR, erythrocyte sedimentation rate; FEV1/FVC, the ratio of forced expiratory volume in 1 second and forced vital capacity; FFS, five-factor score; WBC, white blood cell.

**Supplementary Table 1.** The Prevalence Distributions for All Variables

**Variable**	**Prevalence (%)**	**Variable**	**Prevalence (%)**
ANCA positivity	20.8	Central nervous involvement	0.7
Musculoskeletal involvement	40.8	Respiratory involvement	95.1
Mucocutaneous involvement	51.0	FEV1/FVC < 70%	27.8
Ophthalmologic involvement	13.9	Pulmonary infiltration	72.7
ENT involvement	73.9	Bronchiectasis	31.4
Cardiovascular involvement	60.8	Pulmonary hemorrhage	8.5
Gastrointestinal involvement	53.1	Onset of asthma	50.2
Renal involvement	30.2	Elevated ESR	54.7
Peripheral nervous involvement	61.2	Elevated CRP	62.0

ANCA, anti-neutrophil cytoplasmic antibody; CRP, C-reactive protein; ENT, ear-nose-throat; ESR, erythrocyte sedimentation rate; FEV1, forced expiratory volume in the first second; FVC, forced vital capacity.

**Supplementary Table 2.** The Prevalence of Radiology/Pathology Findings and Inter-Rater Reliability

**Radiology/pathology findings**	**Prevalence (%)**	**Kappa value for agreement between 2 readers**
Bronchiectasis	31.4	0.91
Pulmonary infiltration	72.7	0.93
Vasculitis	29.5	0.86
Granuloma	6.7	0.76
Eosinophil infiltration	52.3	0.91

**Supplementary Table 3.** Fit Statistics for Model 1 from 1 to 4 Classes

**Classes**	**BIC**	*P* of VLMR-LRT*	**Entropy**	**Proportions of classes**
1	2845	—	—	—
**2**	**2822**	**.0110**	**0.567**	**56.3%, 43.7%**
3	2847	.1006	0.645	21.9%, 36.2%, 41.9%
4	2889	.4267	0.696	2.3%, 30.1%, 30.2%, 37.4%

The high diagonal values of the average posterior probabilities (0.85 and 0.89) suggested that the latent classes were well-separated and the model provided a high degree of certainty in classifying individuals.

Information Criteria: Lower value of the BIC indicated a better balance between model fit and parsimony.

Likelihood Ratio Test: The VLMR-LRT was used to compare the model with k classes against a model with k-1 classes. A significant *P*-value (*P* < .05) suggested that the k-class model provided a superior fit to the k-1-class model.

Entropy: Entropy values close to 1 indicated clearer class separation.

BIC, Bayesian information criteria; VLMR-LRT, Vuong–Lo–Mendell–Rubin likelihood-ratio test.

**Supplementary Table 4.** The Characteristics of Patients in 2 Classes of Model 1

	**Class 1**	**Class 2**	***P***
Number	138	107	
Male	79 (57.2%)	66 (61.7%)	.514
Age (years)	53 (39-63)	54 (42-66)	**.017**
FFS	0.88 ± 0.84	0.62 ± 0.79	**.007**
Organ involvements			
Musculoskeletal	88 (63.8%)	12 (11.2%)	**<.001**
Mucocutaneous	104 (75.4%)	21 (19.6%)	**<.001**
Ophthalmologic	18 (13.0%)	16 (15.0%)	.712
ENT	100 (72.5%)	81 (75.7%)	.600
Respiratory	129 (93.5%)	104 (97.2%)	.239
Cardiovascular	115 (83.3%)	34 (31.8%)	**<.001**
Gastrointestinal	98 (71.0%)	32 (29.9%)	**<.001**
Renal	47 (34.1%)	27 (25.2%)	.161
Peripheral nervous	118 (85.5%)	32 (29.9%)	**<.001**
Laboratory findings			
WBC (×10^9^/L)	9.6 (7.6-12.7)	8.5 (11.9-17.8)	**<.001**
Eosinophil (×10^9^/L)	3.79 (1.70-7.81)	1.90 (0.97-4.35)	**<.001**
Lymphocyte (×10^9^/L)	1.85 (1.32-2.61)	1.83 (1.50-2.41)	.703
Hemoglobin (g/L)	128 (114-143)	140 (124-153)	**<.001**
Platelet (×10^9^/L)	245 (173-339)	260 (224-326)	.103
ALT (U/L)	22 (14-42)	18 (13-34)	.168
Urea (mmol/L)	5.2 (3.9-7.2)	4.8 (3.7-6.3)	.266
Creatinine (μmol/L)	72.0 (60.5-85.0)	75.0 (64.0-87.0)	.309
ESR (mm/h)	27 (7-59)	14 (6-42)	**.026**
CRP (mg/L)	10.1 (2.9-47.9)	3.4 (1.2-19.6)	**<.001**
ANCA positivity	25 (18.1%)	26 (24.3%)	.268
Pathological findings			
Vasculitis	35 (36.5%)	9 (17.0%)	**.015**
Granuloma	6 (6.3%)	4 (7.5%)	.744
Eosinophil infiltration	41 (42.7%)	37 (69.8%)	**.002**
Treatments and Outcomes			
Cyclophosphamide	101 (73.2%)	61 (57.0%)	**.010**
Azathioprine	7 (5.1%)	18 (16.8%)	**.003**
Methotrexate	12 (8.7%)	7 (6.5%)	.634
Mycophenolate mofetil	7 (5.1%)	5 (4.7%)	1.000
Follow-up time (d)	5054 (3529-6479)	4495 (2609-6244)	.247
Survival	115 (83.3%)	98 (91.6%)	.084

ALT, alanine transaminase; ANCA, anti-neutrophil cytoplasmic antibody; CRP, C-reactive protein; ENT, ear-nose-throat; ESR, erythrocyte sedimentation rate; FFS, five-factor score; WBC, white blood cell.

**Supplementary Table 5.** Fit Statistics for Model 2 from 1 to 4 Classes

**Classes**	**BIC**	*P* of VLMR-LRT*	**Entropy**	**Proportions of classes**
1	4812	—	—	—
**2**	**4751**	**.0003**	**0.709**	**67.3%, 32.7%**
3	4782	.4166	0.682	27.1%, 38.9%, 34.0%
4	4845	.5561	0.722	8.0%, 26.3%, 28.1%, 37.5%

BIC, Bayesian information criteria; VLMR-LRT, Vuong–Lo–Mendell–Rubin likelihood ratio test.

The high diagonal values of the average posterior probabilities (0.93 and 0.89) suggested that the latent classes were well-separated and the model provided a high degree of certainty in classifying individuals.

**Supplementary Table 6.** The Age and Gender of the Included and Excluded Patients

**Variables**	**Included patients**	**Excluded patients**	***P***
Number	245	32	
Age (years)	59 (41-62)	58 (43-70)	.340
Male (n, %)	145 (59.2)	22 (68.8)	.341

**Supplementary Table 7.** Fit Statistics for Model with Continuous ESR/CRP/Eosinophil Count

**Classes**	**BIC**	*P* of VLMR-LRT*	**Entropy**	**Proportions of classes**
1	10749	—	—	—
**2**	**10645**	**.7642**	**0.887**	**79.2%, 20.8%**
3	10629	.6478	0.766	49.4%, 37.4%, 13.2%
4	10574	.1045	0.837	45.7%, 38.0%, 15.9%, 0.4%

BIC, Bayesian information criteria; VLMR-LRT, Vuong–Lo–Mendell–Rubin likelihood ratio test.

## Data Availability

The data that support the findings of this study are available on request from the corresponding author.
